# Understanding the Effect of Drilling Parameters on Hole Quality of Fiber-Reinforced Polymer Structures

**DOI:** 10.3390/polym16162370

**Published:** 2024-08-21

**Authors:** Katarzyna Biruk-Urban, Paul Bere, Razvan Udroiu, Jerzy Józwik, Karolina Beer-Lech

**Affiliations:** 1 Department of Production Engineering, Mechanical Engineering Faculty, Lublin University of Technology, 20-618 Lublin, Poland; k.biruk-urban@pollub.pl (K.B.-U.); j.jozwik@pollub.pl (J.J.); 2 Department of Manufacturing Engineering, Faculty of Industrial Engineering, Robotics and Production Management, Technical University of Cluj-Napoca, Memorandului 28, 400114 Cluj-Napoca, Romania; paul.bere@tcm.utcluj.ro; 3 Department of Manufacturing Engineering, Transilvania University of Brasov, 29 Eroilor Boulevard, 500036 Brasov, Romania; 4 Department of Mechanical Engineering and Automatic Control, University of Life Sciences in Lublin, 28 Głeboka St., 20-612 Lublin, Poland; karolina.beer-lech@up.lublin.pl

**Keywords:** polymer composites, glass fiber reinforced polymer, drilled hole, thin plate, quality analysis, surface roughness, delamination, statistical analysis

## Abstract

Hole quality in composite materials is gaining interest in aerospace, automotive, and marine industries, especially for structural applications. This paper aims to investigate the quality of holes performed without a backup plate, in thin plates of glass fiber-reinforced polymer (GFRP). The samples were manufactured by two different technologies: vacuum bagging and an innovative method named vacuum mold pressing. Three experiments were designed choosing the control factors that affect the maximum cutting force, delamination factor, and surface roughness of drilled holes in composite materials based on twill fabric layers. Quality analysis of the hole features was performed by microscopy investigations. The effects of the main factors on the targets are investigated using the statistical design of experiments, considering control factors, such as support opening width, weight fraction (wf), feed per tooth, and hole area. The results showed that the feed per tooth and hole area had a more significant influence on the delamination factors and surface roughness (Sa). The best quality of the holes drilled in twill-based GFRP was achieved for a lower feed rate of 0.04 mm/tooth and used a support opening width of 55 mm.

## 1. Introduction

A significant share of the application of fiber-reinforced polymer (FRP) in aerospace and automotive industries, and also in the marine, sport, ship, and medical sectors [1,2] results from their many advantages. The main advantage of FRPs is that the manufacturing technology using lamination techniques is rather easy and accessible. Moreover, FRPs are characterized by excellent unique mechanical properties, good chemical and dimensional stability, and also good corrosion and heat resistance [3]. The most often used fiber-reinforced polymer composite laminates in the industry are carbon fiber (CFRP), glass fiber (GFRP), Kevlar fiber-reinforced polymer materials (KFRP), etc. For example, in the aviation industry, an A-320 aircraft has a 21.5% composite usage to its total weight, and a Boeing 787 and Airbus A350 have 50% of its total weight comprised of CFRP. These parts include the tail cone, center wing box, vertical and horizontal tails, etc. [4]. GFRPs are mainly used indoors, but also in landing gears, fuselage body, tail spoiler, and body [5] which also reduces the weight of the aircraft. Composite materials often require post-processing operations, such as turning [6], drilling [7], milling [8,9], or others to ensure dimensional requirements or to make holes for the assembly process. Machining of FRP composites is extremely difficult due to their inhomogeneous and anisotropic nature, therefore, it is the subject of many research works. Difficult-to-cut fiber reinforcements used in composite materials often are the main cause of an abrasive wear effect on the cutting tool used in machining [10,11]. 

Drilling is a commonly used method for creating accurate holes in various joints such as rivets, especially in the aviation industry. However, drilling causes damage, particularly delamination. Damages caused by drilling are widespread and significantly impact the load-bearing capacity and reliability of components, but also cause assembly tolerance errors [12,13]. In the aircraft industry alone, drilling-induced delamination is responsible for approximately 60% of part rejections during the final assembly. To prevent material failure, it’s essential to use appropriate tools and optimal drilling conditions [14]. 

The most common types of damage, besides delamination, caused by the machining of GFRP composites are fiber pullouts, interlaminar delamination, fiber/matrix debonding, fuzzing, matrix melting, and softening [15]. Delamination is caused by a combination of two mechanisms: mechanical and thermal damage, and it occurs when the tool exits the composite material due to feed forces. Delamination during machining can occur in two types: peel-up delamination and push-out delamination. Peel-up delamination occurs at the top surface of the composite materials when the upper layer fibers are not properly cut due to improper machining conditions and when the cutting edges of the tool touch the laminate. The peeling force generated by the slope of a drill flute removes the top layers, causing peel-up delamination [16]. Push-out delamination occurs at the down surface of the machined composite, as the drilled composite is subjected to axial and bending forces [17]. As it was found in previous research on FRP composites drilling the push-out delamination is more critical than the peel-up [18,19] and is related to technological parameters of drilling [20,21]. 

The quality of the drilled holes is strongly influenced by the possible damages that take place due to composite unique characteristics such as anisotropy, non-homogeneity, and abrasive and hard-reinforced fibers. The main quality attributes of the drilled holes are hole size, circularity, delamination, surface roughness, and heat-affected zone. These characteristics are investigated by several researchers to find the optimum combination of input factors to achieve good-quality holes in drilled FRPs.

The primary control factors that affect the surface quality of the holes drilled in laminated composite materials are the cutting speed, drill tool type, feed per tooth, laminate thickness, weight fraction, influence of the use of the drill support, specific areas of the hole and particular manufacturing features. There are some experimental design approaches to statistically investigate the influence of control factors on a target, e.g., factorial design and Taguchi method. Some researchers used this method to investigate the quality of the drilled holes in composite materials. Malik et al. [22], using factorial design, investigated the drilling performance of GFRP composite, based on the thrust force, temperature, and delamination factor, and concluded that the best drilling performance was achieved by the solid carbide tool at a low feed rate. Taguchi method was used by Ngah et al. [23] to investigate the influence of process parameters such as spindle speed, feed rate, type of drill bits, and geometry on the delamination of drilled holes in kenaf-glass fiber-reinforced unsaturated polyester composite. One of the conclusions was that the quality of the drill hole could be improved using a twist drill bit.

Some research has shown that the optical roughness measurements are less sensitive to measurement position than the stylus, and increase the accuracy of roughness measurement for machined FRP surfaces [24]. Kim et al. [25] using a CFRP workpiece consisting of 40 layers of IM7carbon fibers with an epoxy matrix in the quasi-isotropic layups, and a backup aluminum plate with predrilled holes 3 mm larger than the drill’s nominal size, investigate the hole quality in terms of hole size and surface roughness. Using a contact profilometer, its stylus could not reach the narrow area where the deepest fiber pullout happened at the plies, resulting in some limitations of the measurements.

There are two different aspects regarding the drilling scheme of thin composite plates, backup drilling and drilling without the backup. Backup drilling or supported drilling supposes the placement of a backup plate under the specimen, while in unsupported drilling or drilling without the backup, there is nothing under the specimen. Some researchers conducted research on the influence of the use of support during drilling as an important factor from the point of view of delamination. Gemi et al. [26] in their research on the drilling of different GFRP composite pipes tested the influence of different feed rates and back support on the thrust force, which influences the delamination. They also compared results with unsupported samples. It was found that the use of back support significantly increases the thrust force by 3 to 35%. Moreover, an increase in feed rate caused an increase in thrust force, and lower values of thrust force were obtained for cases without backup support. Tsao et al. [27] examined how backup plates affect delamination when drilling composite materials with saw drills and core drills. Using the critical drilling thrust force it was calculated and compared with cases without backup at the onset of delamination. Based on the results both the saw drill and the core drill with backup generate a critical thrust force than those without backup. Compared to industrial experience, the results of this study show that the drill can be operated at a higher feed rate without damaging the delamination. Heidary et al. [28] drilled composite samples with an HSS twist drill with and without support with different feed ranges (0.25 to 1.16 mm/r). It was found that the delamination factor of the supported specimens was decreased in the range of 1.8% to 20.7% compared to those drilled without backup support. Also in the research, [29] the influence of the exit support plate (5 mm thick placed under the sample) and drilling parameters (feed rate, cutting speed, and drill point angle) on delamination in twist drilling of GFRPs was analyzed. It was found that delamination decreased in the range of 8–27% when using a backup plate under the composite specimen depending on the drilling parameters. The influence of support on multi-hole drilling for GFRP composite materials was examined in [30]. The authors focused on thrust forces and exit delamination damage when two types of support were applied during drilling (round-hole array backing plate and square-hollow backing plate). For support in the form of a round-hole array backing plate, the value of thrust forces and exit delamination was constant and lower than for a square-hollow backing plate. Ciecielag in the research [31] examined the influence of the GFRP and CFRP samples’ stiffness on the accuracy of drilled holes and delamination in the drilling process. The tests were performed with constant drilling parameters but the length of unsupported elements was various. The experiments found that the feed force increases with the increase in the length of the element for GFRP and CFRP samples. The length of unsupported elements also influences the accuracy of the drilled holes.

Knowledge of the mechanical properties of new composite materials that can be used, e.g., in aviation, is particularly important from the point of view of flight safety. Therefore, researchers conduct research on mechanical properties. Ya-Jung Lee et al. [32,33] applied in their tests polyester resin with glass-fiber-reinforced fillers for tests of mechanical properties (tensile strength, flexural strength, and Young’s modulus) for single and multiple fibers. They found that the vacuum infusion processed GFRP samples had better mechanical properties than the hand layup technique, which increased the porosity of those composites. Jesthi and Nayak [34] based their research on improving the mechanical properties of marine application-based fiber-reinforced composite materials by hybridizing glass and carbon fibers. The research [35] focuses on the influence of stacking sequence on the strength of hybrid composites composed of materials with varying stiffness and strength. It was found that hybrid composite laminates containing 50% carbon fiber reinforcement have the best flexural properties with carbon layers on the outside, while the alternating carbon/glass layup has the highest compressive strength. According to the findings, the stacking sequence has no effect on tensile strength.

From the survey in the literature, the following results were found:

- There are few studies about drilling without a backup. The process of drilling without support is often used when drilling by robots of thin composite components that should be riveted, in the aerospace field.
- There are many factors affecting the surface quality of the holes drilled in laminate composites, meaning delamination and surface roughness. The effect of drilling factors on the hole quality of fiber-reinforced polymer structures should be analyzed and understood for implementation in the industry.
- There is a necessity to determine the mechanical characteristics of new composite materials which will be machined.

The main aim of this article is to investigate the quality of drilled holes, in very thin plates of glass fiber-reinforced polymers, drilled without a backup plate, the specimens being clamped from both sides during drilling operations, at different opening widths. The composite plates are manufactured by two different technologies, an innovative one called vacuum mold pressing and the well-known technology of autoclave vacuum bagging.

The novelty of the research presented in this study lies in a methodology that combines experimental design and statistical analysis to understand the effect of drilling parameters on the hole quality of fiber-reinforced polymer structures. Thus, the mechanical properties of a GFRP/epoxy composite, the delamination during drilling with different support widths, and the surface roughness of the drilled holes were investigated. 

## 2. Materials and Methods

A methodology regarding the investigation of the quality of holes made in composite materials was proposed. The methodology consisted of several steps such as the design of experiments, manufacturing and machining of the samples, determination of the mechanical properties of the samples, measurement of delamination and surface roughness, and statistical analysis of the hole quality characteristics.

**Figure 1:**
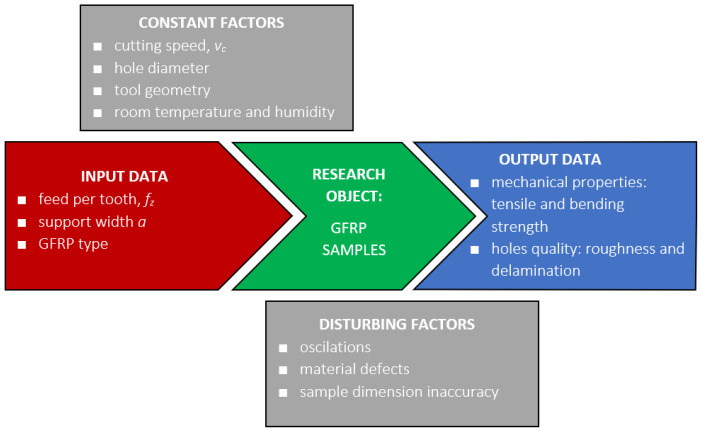
Experimental plan.

This study aimed to examine how specific technological drilling parameters, such as feed per tooth (*f_z_*) and the width of the drilled sample affected the maximum cutting force, delamination, and surface roughness of the holes in various types of GFRP materials. The analyzed materials differ by wf ratio and technological manufacturing process. The goal was to obtain drilled holes of very high quality. The general experimental plan is presented in Figure 1.

### 2.1. Materials and Manufacturing Process

The selection of the control factors depends on the particularities of the manufacturing technology, the material characteristics, and the specific zone of the hole. Optimization of the cutting speed was preliminarily made in a previous study [36]. An optimal drill tool for laminated composite materials is a carbide drill, as is mentioned in [22]. There were no studies about the influence of support width on the surface quality of the drilled hole found. Thus, support width, feed per tooth, weight fraction, and hole zone were chosen as the critical factors used for the surface quality analysis (delamination and surface roughness).

To carry out the research, GFRP plates were used and were drilled using different technological parameters. GFRP plates were used to more easily visualize the defects that appeared during the process of drilling and delamination of the layers. The GFRP plates after manufacturing were transparent so that any defect could be easily detected. 

Several plates marked with A1 to A4 from GFRP were manufactured. A 2 *×* 2 Twill fabric of 280 g/sq from glass fibers was used as reinforcement material. An Epikote MGS LR135/LH 136 type epoxy matrix (Lange&Ritter GmbH, Gerlingen, Germany) was used for impregnation. For the plates marked with A1-A2-A3, 4 layers of twill fabric were used, and for the plates marked with A4, 8 layers were used. Stacking sequences were [0/90]_4_ and [0/90]_8_, respectively, for the boards marked with A4.

**Figure 2:**
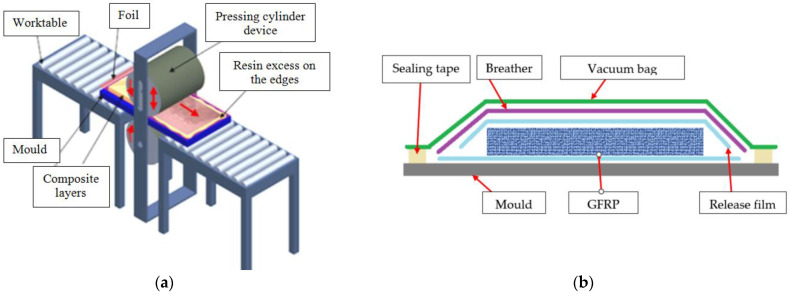
Schematic representation of the sample manufacturing: (**a**) vacuum mold pressing (technology 1) [37] for plates noted A1–A3 (**b**) vacuum bag forming (technology 2) for plates noted A4.

Wet impregnation technology was used for all types of plates. For the plates marked with A1–A3, an innovative composite plate manufacturing technology (Figure 2a) was used (technology 1) and presented in detail in [20,36,37]. The process involved the impregnation of the layers using the hand layup technology of the layers using a flat metal mold. At the end, the GFRP layers are covered with 3 µm-thick Mylar (Polyester film). In the stage where the resin is still unpolymerized (fluid) but after it has passed the gel time, the mold, together with impregnated GFRP and covered with foil, is passed through an installation like a calendar with two cylinders. In this way, the composite is pressed and the excess resin is pushed towards the edges of the composite. Once the resin is removed from the composite, the air bubbles from the composite are also removed.

Thus, the resin on the edges seals the surface between the mold and the composite. Due to the viscosity of the resin, air no longer enters the composition of the composite, resulting in a pressed composite without air bubbles in the board structure. When pressing and removing the excess resin, the volume of the composite decreases. In this way, together with the reduction of the material volume and the sealing of the edges between the foil and the mold, a vacuum pressure is produced in the material that keeps the composite material free of air bubbles. Pressing the GFRP plates with different cylinder forces resulted in obtaining different wf intentionally, in order to evaluate the behavior of these materials in the drilling process without support. The curing process for plates produced by technology 1 consists of treatment at 22 °C for 24 h followed by a heat treatment at 80 °C for 8 h.

A different method of vacuum bag forming, and autoclave curing was used to manufacture the plates marked with A4 (technology 2). The procedure and the technological parameters used in the autoclave are presented in detail in [38] and include the hand layup of the layers and autoclave curing procedure (Figure 2b). The Maroso autoclave (Maroso SRL, Pianezze, Italy) curing procedure consists of some steps. The temperature was increased from 0 °C to 80 °C in 30 min, applying a pressure of 4 bars. Then, the temperature was increased from 80 °C to 120 °C in 20 min and the pressure was kept at 4 bars. In the third step of the cycle, the temperature was kept at 120 °C and the pressure at 4 bars for 2 h. At the end of the cycle, the pressure was reduced to 0 bars, and the temperature was decreased to 60 °C in 30 min. This procedure is used to manufacture aviation components from FRP and the results obtained in the evaluation of the materials or the behavior of the materials in different processing can be used for other composite materials. The properties of the manufactured materials are presented in Table 1.

### 2.2. Drilling Methodology

**Figure 3:**
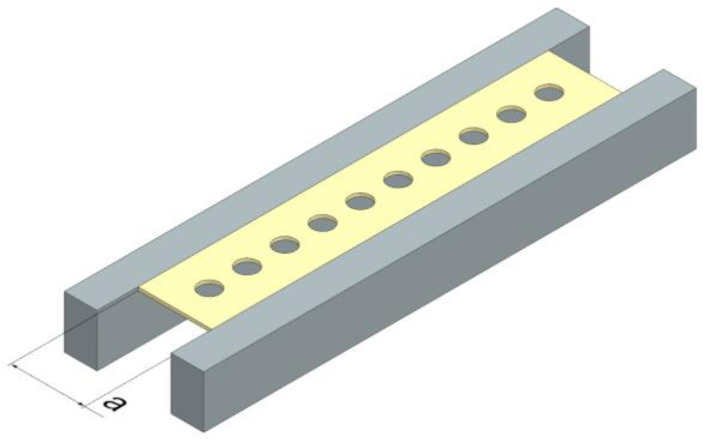
Scheme of mounting samples clamped in a vice, using different support opening width “a”, where *a* = 45, 55, 65, or 75 mm.

A Waterjet Combo (Legnica, Poland), which is an abrasive water jet cutter, was employed to cut GFRP plates into drilling samples. The samples differed in width (Figure 3), were 250 mm long, and the thickness varied based on the composite type. The samples were clamped in a vice during processing. Each sample was drilled with a total of 10 holes, spaced 25 mm apart along the hole axis.

The drilling process was carried out using a vertical machining center, specifically the Avia VMC800HS (Avia, Warsaw, Poland), without the use of coolant. The drilling was conducted with a 2-edge carbide diamond-coated drill with a diameter of 12.726 mm, specifically the SD205A-12.726-56-14R1-C2 model (Seco, Erkrath, Germany). Throughout the experiments, holes were drilled in samples employing varying feeds per tooth (*f_z_*) of 0.04, 0.08, 0.12, and 0.16 mm/tooth and a constant cutting speed *v_c_* = 182 m/min. The selection of technological parameters was preceded by preliminary tests.

One of the measurements performed during the research was the measurement of cutting forces during the drilling process. The stand for measuring cutting forces in three axes *F_x_*, *F_y_*, *F_z_* during drilling consisted of: a 9257B dynamometer from Kistler (Winterthur, Switzerland), a signal conditioning system, a DAQ module with an integrated A/D card, and dedicated DynoWare software V 5.0 (DynoJet, Germany) for recording curves. The research focused on the results of the maximum feed force *F_z_*, which played a major role in the drilling process. For a high-quality hole and process improvement, it is important to monitor and control cutting forces during drilling.

### 2.3. Mechanical Properties Methodology

#### 2.3.1. Tensile Strength Tests

To determine the tensile properties of various types of GFRP samples, the specimens underwent a static tensile test. In this research, for analyzing the mechanical behavior of the glass fiber composites, the specimens were tested using the servo-hydraulic testing machine Instron 8801 Dual Column (Instron, Norwood, MA, USA). Tensile tests were conducted following the ISO 527-5 standard, using type A samples. Samples with bonded tabs had the following dimensions: overall length—*L*_1_ = 250 mm, width—*b* = 25 mm, distance between the end tabs—*L*_2_ = 150 mm, and the thickness *h* varied based on the GFRP type. Five samples were performed for each of the GFRPs tested. The tests were performed at the environmental temperature of 23 ± 3 °C, and relative air humidity of 50 ± 5%. The specimens were loaded with a constant speed of 2 mm/min until breaking.

The tensile strength was calculated as:

(1) $$R_{m}=\frac{F_{m}}{S_{0}}$$

where: $F_{m}$ is the maximum tensile force, and $S_{0}$ is the specimen cross-sectional area.

#### 2.3.2. Bending Strength Tests

In order to determine the bending strength of various types of GFRP samples, three-point bending tests were conducted using a testing machine. Bending tests were conducted following the EN ISO 14125 standard, using type A samples. Samples had the following dimensions: overall length—*l* = 60 mm, length of the span supports—*L* = 40 mm, width—*b* = 10 mm, and the thickness—*h*, which varied based on the GFRP type. Five repetitions were performed for each variable. The tests were performed at an environmental temperature of 23 ± 3 °C and a relative air humidity of 50 ± 5%. The specimens were loaded with a constant speed of 5 mm/min until breaking. The bending strength was calculated as

(2) $$\sigma_{f}=\frac{3FL}{2bh^{2}}$$

where F is the axial load (force) at the fracture point, L is the length of the support span, b is the sample width, and h is the sample thickness.

### 2.4. Hole Quality Analysis Methodology

This study aimed to analyze the quality of the drilled holes, on the top and bottom surfaces. There are three main parameters that can characterize the quality of the holes, delamination, hole circularity and surface roughness. In this study, delamination analysis and surface roughness analysis was taken into consideration.

**Figure 4:**
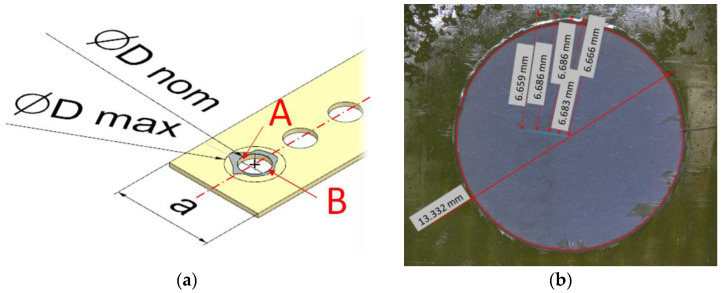
Determination of the delamination factor and hole surface roughness: (**a**) measurement method, (**b**) five measurements using an optical microscope.

Two types of delamination were identified in this research. The first one, peel-up delamination occurs on the top surface and the second one, push-out delamination occurs on the down surface of the drilled sample when the drill exits the material. The delamination was quantified using the delamination factor F_d_ (Figure 4). It is the ratio of the maximum diameter to the nominal diameter and is expressed by the following formula [39]:

(3) $$F_{d}=\frac{D_{max}}{D_{nom}}$$

where $F_{d}$ is the delamination factor, $D_{max}$ is the maximum delaminated diameter drawn from the centerline of the hole, and $D_{nom}$ is the nominal diameter.

The diameters of peel-up and push-out delamination were measured using the software provided with the Keyence VHX-5000 (Osaka, Japan) optical microscope at 20× magnification. For each hole, a photo was taken on a microscope and the five maximum delamination diameters were measured on the upper and lower surfaces of the sample. The maximum delaminated diameter was drawn from the centerline of the hole’s nominal diameter to the point where the largest area of delamination was observed. The average value was determined from the five values and was considered during the analysis of the results.

Longitudinal cuts through the center of the holes were performed, in order to prepare the samples for roughness measurements, as is shown in Figure 4a. It resulted in two halves, called A and B. Three surface roughness measurements were performed for each half of the hole of each specimen. Alicona Infinite Focus G5 (Raaba, Graz, Austria) optical surface roughness device, was used for the measurement of 3D surface roughness parameters. In these measurements, the sampling area was set as 2.25 × 1.50 mm^2^, and a cutoff parameter of 0.8 mm. For the purposes of this article, the surface roughness Sa parameter was selected for analysis. Sa parameter expresses, as an absolute value, the difference in height of each point compared to the arithmetical mean of the surface. The average roughness Sa was calculated for each hole.

### 2.5. Design of the Experiments (DOE) and Statistical Analysis

Three experiments were designed choosing the control factors that affect the targets. The following targets were considered: delamination factor, maximum cutting force, and surface roughness of drilled holes in composite materials. Three general full factorial designs with 48-, 24-, and 32-factor combinations, were performed to be able to investigate the influence of the factors on targets for the three experiments.

In the first experiment, with the peel-up and push-out delaminations as targets, the support width, weight fraction of the composite material, and feed per tooth as control factors were considered for materials made by technology 1 (A1–A3), as is shown in Table 2. 

Maximum cutting force was taken as the target for the second experiment and the same control as the previous experiment was kept. In the third experiment, surface roughness (Sa) as a target, and support width, weight fraction, and hole zone as control factors were taken into consideration as is shown in Table 3. The control factors used for the design of the experiments for material made by technology 2 (A4) are presented in Table 4.

Statistical analysis allowed the investigation and characterization of the effects of control factors and their interactions on each target.

The contributions of the control factors to the maximum cutting force, Fd_peel-up, Fd_push-out, and Sa were determined using generalized linear models (GLM) analysis within the Minitab 19 software (Coventry, UK). The generalized linear model is a more general approach to performing an analysis of variance (ANOVA) [40]. The significant factors were determined from the ANOVA table, for all the DOE. Thus, the F-values and the *p*-values were analyzed in order to make a decision concerning the statistical significance. Also, the percentage contribution ratio (PC%) of each factor and interactions between factors were determined. 

Graphical methods were used to evaluate the influence of control factors on target factors. Also to explain the statistical results, three types of graphs, the main effects plot, interaction effects plot, and interval plot of target versus control factors, were used.

ANOVA assumptions were checked and validated as follows: residuals should be normally distributed, the variance of the observations in each treatment should be equal, and the response should be independent and identically distributed [41].

## 3. Results and Discussion

Results are focused on the mechanical properties of the materials, cutting force analysis, delamination analysis, and surface roughness analysis of the holes.

### 3.1. Mechanical Properties

**Figure 5:**
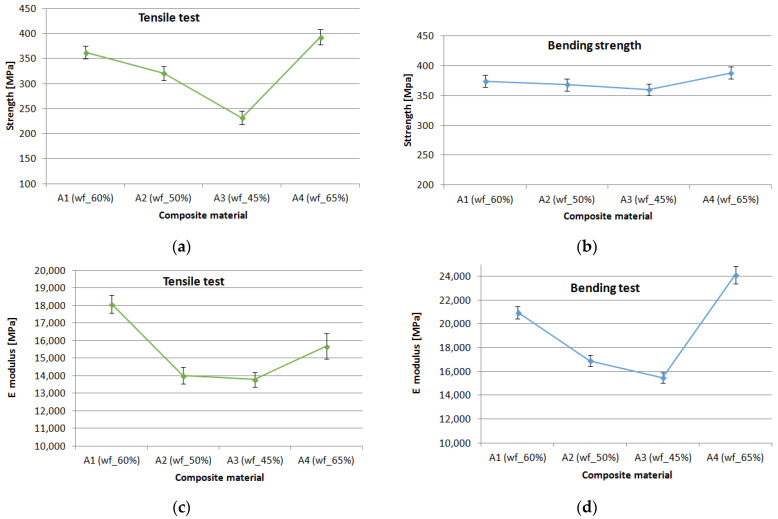
Mechanical properties of composite samples: (**a**) tensile strength; (**b**) bending strength; (**c**) tensile modulus; (**d**) bending modulus.

The results of the mechanical properties of the materials A1–A4 are shown in Figure 5. 

The presented results in Figure 5a–d regarding the mechanical characteristics of the tensile and bending tests of specimens A1–A4 highlight a specific behavior of GFRP. Both the breaking strength and the modulus of elasticity increase with the increase in wf ratio of the composite. The results of the statistical analysis have shown coefficients of variation lower than 10% for the set of five tested samples, which indicates a good repeatability of the data. This confirms the homogeneity of the tested material and the accuracy of the manufacturing processes used for the different GFRP plates.

### 3.2. Cutting Force

**Figure 6:**
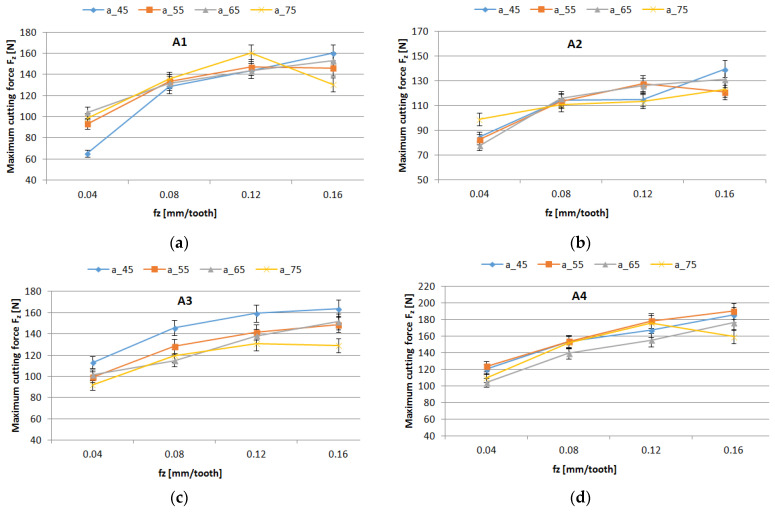
Influence of the feed per tooth on Cutting force factor for different support widths: (**a**) material A1; (**b**) material A2; (**c**) material A3; (**d**) material A4.

The trends of maximum cutting force versus the support width, and feed per tooth, for different material types (wf), are shown in Figure 6.

The results of the statistical analysis of maximum cutting force are shown in Table 5. The sequential SS (Seq SS) shows the variation in control factors and determines the significance of terms by the order in which they enter the model. It results that the weight fraction and the feed per tooth had the highest influence on the maximum cutting force, as long as the *p*-value was lower than the significance level of 0.05. The most significant percentage contribution ratios were obtained for feed per tooth and weight fraction. The most significant factor in the cutting force was the feed per tooth, which explained 62.15% of the total variation for technology 1 (materials A1–A3) and 92.42% for technology 2. 

**Figure 7:**
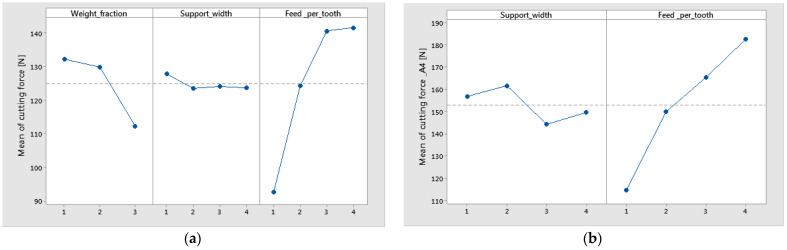
Main effects plot for cutting force factor: (**a**) material A1–3 (technology 1); (**b**) material A4 (technology 2).

**Figure 8:**
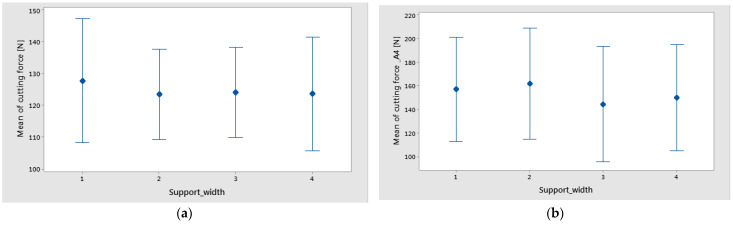
Interval plot of cutting force factor with support width: (**a**) technology 1; (**b**) technology 2. Individual standard deviations were used to calculate the interval plot. Bars are standard errors of the mean.

**Figure 9:**
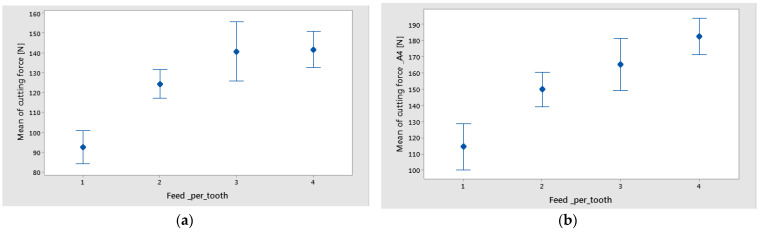
Interval plot of Cutting force factor with feed per tooth: (**a**) technology 1; (**b**) technology 2 Individual standard deviations were used to calculate the intervals plot. Bars are standard errors of the mean.

The statistical results were plotted, resulting in the main effects plot (Figure 7), and interval plot (Figure 8 and Figure 9) of cutting force versus control factor. It can be seen, in the case of technology 1, that the main effects of cutting force were the weight fraction at level 1 (60%), the support width at level 1 (45 mm), and the feed per tooth at level 4 (0.16 mm/tooth), as is shown in Figure 7a. Accordingly, for the material containing a higher value of the wf ratio of reinforced material, drilling forces to break through the fibers are higher than for the composite with a lower wf. Increased fiber volume fraction results in increased thrust forces. Similar results were obtained for technology 2, with the note that the support width at level 2 (55 mm) had the greatest impact on cutting force, as is shown in Figure 7b. 

The graphs from Figure 8 and Figure 9 show the interval plots with standard error bars of each factor versus the cutting force. While the means appear to be different, the difference in cutting force in the support width was probably not significant because all the interval bars easily overlapped (Figure 8a,b). The mean values of cutting force for material A4 are higher for materials A1–A3 (Figure 9).

It is important to monitor the cutting forces during drilling for a high-quality hole and process improvement. Thus the results from this study have shown the following:

- For drilled holes in thin plates, the support width factor has a low contribution to the maximum cutting force. However, the mean lowest value of cutting force was obtained for the support width of 45 mm.
- It was observed that the mean of maximum cutting force has increased with the increase in thickness of the plate (Figure 8).
- The most significant percentage contribution ratios were obtained for feed per tooth and weight fraction.

### 3.3. Holes Quality Analysis 

The study has analyzed the quality of the drilled holes, on the top and bottom surfaces, taking into consideration the main parameters that can characterize the quality of the holes, such as delamination, and surface roughness. The results were presented in the next subsections.

#### 3.3.1. Delamination Analysis 

**Figure 10:**
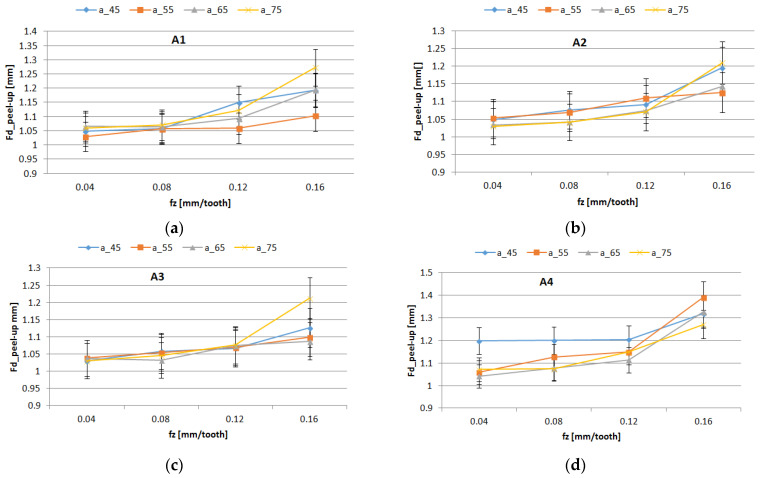
Influence of the feed per tooth *f_z_* on Fd_peel-up factor for different support width: (**a**) A1 material; (**b**) A2 material; (**c**) A3 material; (**d**) A4 material.

**Figure 11:**
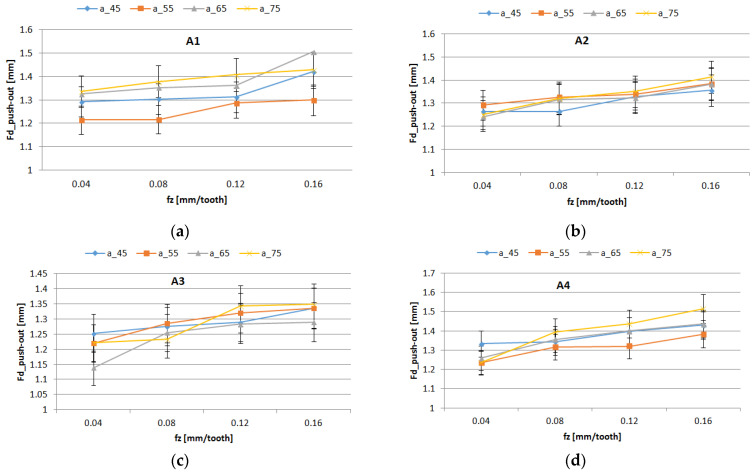
Influence of the feed per tooth *f_z_* on Fd_push-out factor for different support widths: (**a**) A1 material; (**b**) A2 material; (**c**) A3 material; (**d**) A4 material.

The experimental curves of the delamination factors distribution Fd_peel-up and Fd_push-down, for all studied materials and are shown in Figure 10 and Figure 11. The curves present similar trends. The highest value for delamination factors was obtained for Fd_push-down.

The results of the ANOVA analysis for delamination factors are shown in Table 6. The Feed_per_tooth, Weight_fraction, and support width had a more significant influence on the Fd_peel-up and Fd_push-out delamination factors as long as the *p*-value was lower than the significance level of 0.05. 

The most significant influence on the delamination was the feed per tooth, which explained 69.14% (Fd_peel-up) and 45.97% (Fd_push-out) of the total variation, respectively, which is also confirmed in different papers [42]. The support width and weight fraction factors had a lower percentage contribution, as is shown in Table 6. A higher contribution of the weight fraction of 29.67% was obtained for Fd_push-out. The interactions Support_width*Feed _per_tooth and Support_width*Weight_fraction showed a significant effect on Fd_peel-up. A significant effect on Fd_push-out was observed for Feed_per_tooth, Weight_fraction, and the second-order interaction Weight_fraction*Feed_per_tooth. The R-squared values of 97.28% for Fd_peel-up and 93.13% for Fd_push-out have indicated that the model explains all the variability of the response data around its mean.

**Figure 12:**
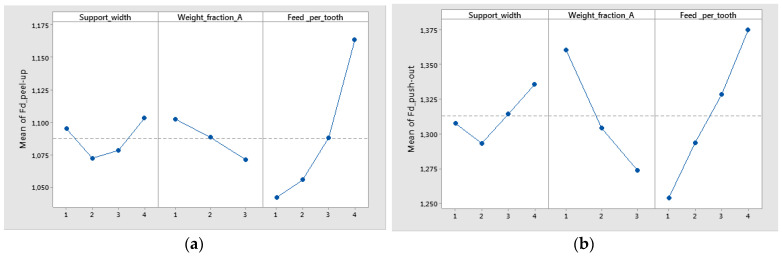
Main effects plot for delamination factor for twill-based materials (A): (**a**) Fd_peel-up; (**b**) Fd_push-out.

The influence of control factors on delamination was statistically evaluated using graphical methods. The main effects plot for delamination, interaction effects plot for delamination, and interval plot of delamination versus the control factors were plotted. The results show that support width at level 4 (75 mm), weight fraction at level 1 (60%), and Feed_per_tooth at level 4 (0.16 mm/tooth) had the main effects plot for delamination (Figure 12). The minimum mean values of Fd_peel-up and Fd_push-out factors were obtained for 55 mm width support (Figure 12). The lower values of the support width improve the stiffness of the sample and reduce the deflection during drilling causing the reduction of delamination.

**Figure 13:**
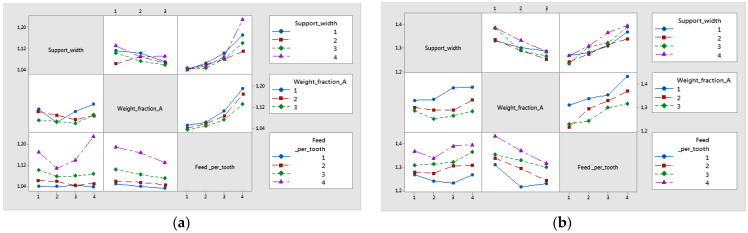
Interaction effects plot for delamination factor for twill-based composite materials A1–A3 made of technology 1: (**a**) Fd_peel-up; (**b**) Fd_push-out.

Figure 13 displays an interaction plot matrix for the mean of delamination factors, which shows no interaction for parallel lines, and shows interaction for intersecting lines. The interaction of Support_width with Feed_per_tooth had a significant influence on the delamination factor, as shown in Figure 13.

**Figure 14:**
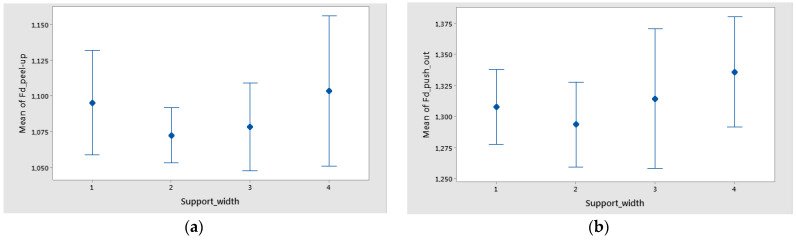
Interval plot of support width in the case of twill-based composite materials A1–3 made of technology 1 for: (**a**) Fd_peel-up; (**b**) Fd_push-out. Individual standard deviations were used to calculate the interval plot. Bars are standard errors of the mean.

**Figure 15:**
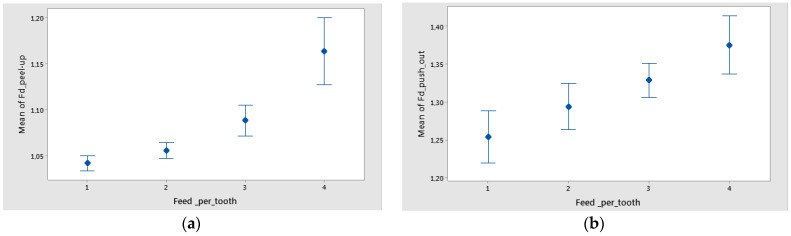
Interval plot of feed per tooth in the case of twill-based composite materials A1–3 for the following: (**a**) Fd_peel-up; (**b**) Fd_push-out. Individual standard deviations were used to calculate the interval plot. Bars are standard errors of the mean.

**Figure 16:**
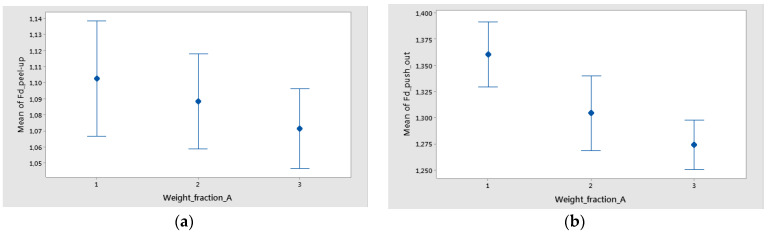
Interval plot of weight fraction in the case of twill-based composite materials (A1–A3) for the following: (**a**) Fd_peel-up; (**b**) Fd_push-out. Individual standard deviations were used to calculate the interval plot. Bars are standard errors of the mean.

The interval plots with the standard error bars of each factor versus delamination factors Fd_peel-up and Fd_push-out are shown in Figure 14, Figure 15 and Figure 16. 

The mean of Fd was higher for a support width of 75 mm and lower for a support width of 55 mm (Figure 14). The difference between the means for delamination in Feed_per_tooth, was significant because the interval bars did not overlap (Figure 15).

The mean of Fd was higher for push-out (Figure 16). Also, only for Fd_push-out the difference between the means for delamination in weight fraction was significant because the interval bars did not overlap, as is shown in Figure 16b.

**Figure 17:**
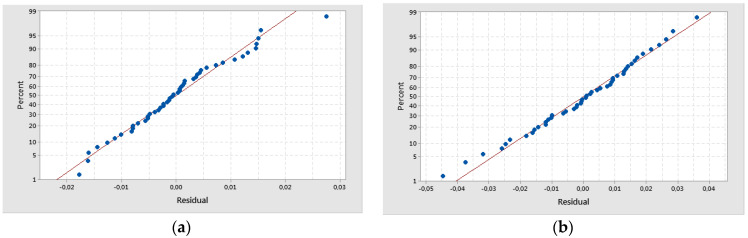
Normal probability plots of delamination factor: (**a**) Fd_peel-up; (**b**) Fd_push-out.

The generalized linear models were checked for model adequacy by using the normal probability plots of residuals [41,43], which resulted in a normal distribution, as is shown in Figure 17. Also, normal probability plots of residuals were found for all the experiments.

The results of the statistical analysis for delamination factors in the case of material A4 made of technology 2 have shown that Fd_zone and Feed_per_tooth are the most significant factors. Support width had a lower influence on delamination. The highest contribution on delamination was obtained for Fd_zone with a percentage contribution (PC%) around 54.51%, as is shown in Table 7.

**Figure 18:**
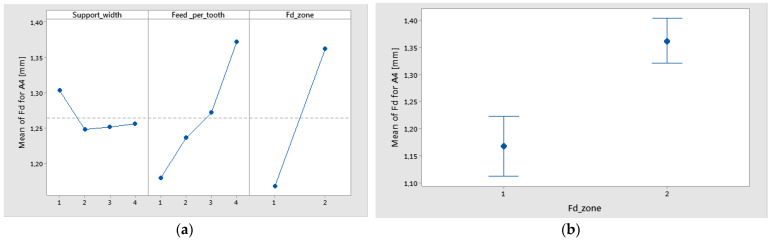
(**a**) Main effects plot for delamination factor for A4; (**b**) Interval plot of Fd; Fd_peel-up (1) Fd_push-out (2). Individual standard deviations were used to calculate the interval plot.

**Figure 19:**
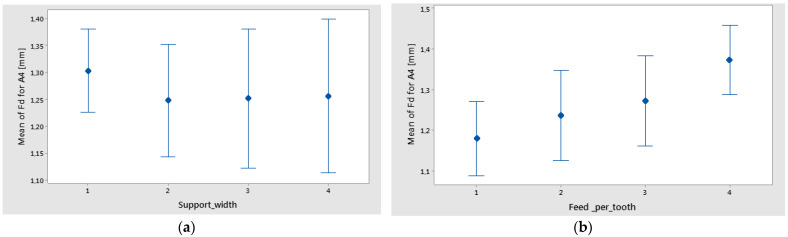
Interval plot of Fd for A4 material: (**a**) support width; (**b**) feed per tooth. Individual standard deviations were used to calculate the interval plot.

The statistical results were plotted, resulting in the graphs from Figure 18 and Figure 19. From Figure 18a, it can be seen that the main effects of the delamination factor of material A4 were the support width at level 1 (45 mm), Feed_per_tooth at level 4 (0.16 mm/tooth), and the Fd_zone at level 2 (push out). The interval plot with standard error bars of Fd_zone, support width, and feed per tooth versus the delamination factor (Fd) are shown in Figure 18b and Figure 19.

#### 3.3.2. Surface Roughness Analysis

**Figure 20:**
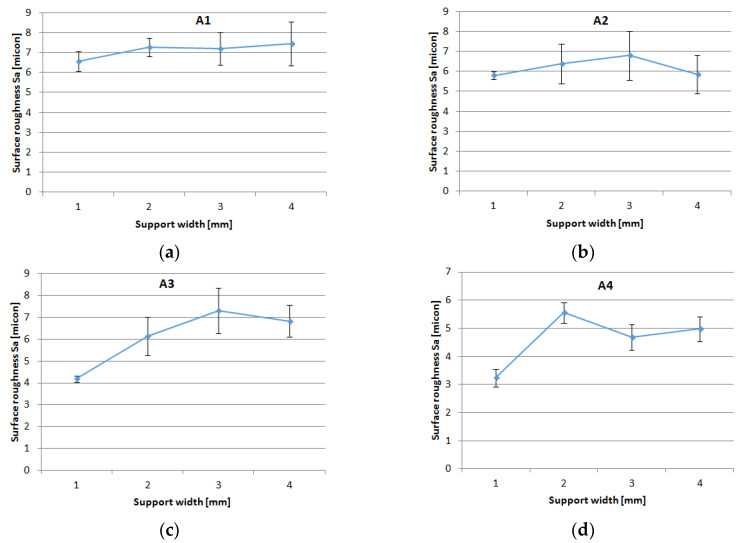
Influence of the feed per tooth on Sa for different support widths: (**a**) A1 material; (**b**) A2 material; (**c**) A3 material; (**d**) A4 material.

The surface roughness of the holes was analyzed in two distinct areas on the circumference of the hole. The experimental curves for the mean values of surface roughness (Sa), are shown in Figure 20. The trends of Sa versus support width showed an increase in Sa with the increase of support width. 

From Table 8, it can be seen that the hole area, support width, and weight fraction had a higher significant influence on the Sa because the *p*-value is lower than 0.05.

**Figure 21:**
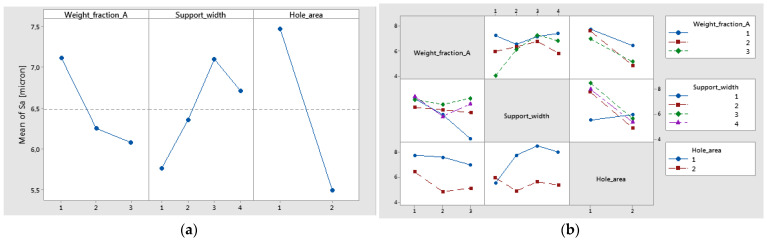
(**a**) main effects plot for Sa; (**b**) interaction plot of Sa.

The most significant factor on the roughness parameter (Sa) was the hole area, with a contribution of 39.72% of the total variation. The next largest contribution on Sa came from the interaction between support width and hole area, with a contribution of 20.03%. This is also confirmed by the interaction plot of Sa (Figure 21b) shown in the case of intersecting lines.

**Figure 22:**
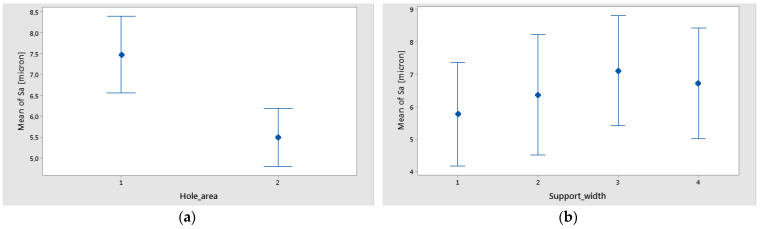
Interval plot of Sa for the following: (**a**) hole area; (**b**) support width. Individual standard deviations are used to calculate the intervals.

It can be seen (Figure 21a) that the main effects of Sa were the weight fraction at level 1 (60%), the support width at level 3 (65 mm), and the hole area at level 1 (A zone). The interval plots of Sa for hole area and support width are shown in Figure 22a,b.

The Sa has a decreasing trend depending on the decrease in weight fraction. Thus, the lowest roughness resulted in the A3 material. This is explained by the fact that this material contains more resin and the layers are thicker.

**Figure 23:**
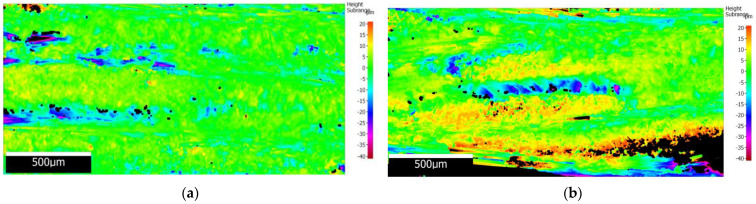
Surface topography map of roughness for the representative holes: (**a**) low roughness A3-45 mm-zone A; (**b**) high roughness A1-65 mm-zone B.

From the main effect plots graphic (Figure 21a), it was found that lower mean roughness was obtained for A3 material with 45 mm width, and higher roughness for A1 material with 65 mm width, as shown in Figure 23.

As can be seen in the results obtained regarding the roughness of the drilled surface in the GFRP plates, with the parameters used, wf has an influence on the surface quality; the lower wf, the more Sa decreases. From the presented images of the surface topography map, you can see the yellow areas that represent the monofilaments grouped in threads that are cut transversely by the drill. These areas determine a higher obtained roughness. In Figure 23a where wf is lower and the monofilaments are rarer (higher amount of polymer), the surface roughness also decreases. This conclusion can predict the Sa results obtained in the case of using several layers of the reinforcing material arranged transversely, which in the case of drilling will have a higher roughness. With the number of layers of the reinforcement material, they will be arranged unidirectionally and Sa will be higher.

## 4. Conclusions

This paper investigated the quality of holes drilled in composite materials plates having different wf, and made by two different technologies. The drilling process was done on the GFRP composites using various main input parameters such as support width, wf ratio, and feed per tooth. The maximum cutting force, delamination factor (at entry and exit), and internal surface roughness were examined. The following conclusions can be drawn:

- There is a need for the surface quality of holes in composite materials manufactured parts in the industry.
- For a high-quality hole and process improvement, it is important to monitor and control cutting forces during drilling. For drilled holes in thin plates, the support width factor has a low contribution to the maximum cutting force. However, the mean lowest value of cutting force was obtained for the support width of 45 mm.
- The results showed that the cutting force, and delamination factors (Fd_peel-up, Fd_push-out) could be minimized significantly by reducing the feed per tooth.
- The highest Fd was found for the support width of 75 mm, and the lowest was for the support width of 55 mm, which showed the advantage of using a smaller support width for GFRP composite.
- The most significant influence on the delamination factor was the feed per tooth of 69.14% for Fd_peel-up, respectively 45.97% for Fd_push-out. The support width and weight fraction factors had a lower percentage contribution. A higher contribution of the wf of 29.67% was obtained for Fd_push-out. The interactions Support_width with wf showed a significant effect on Fd_peel-up.
- It was determined that the support width contribution has decreased with the increase in thickness of the plate. It can be concluded that for thin plates, the support width has a significant effect.
- The highest surface roughness (Sa) was found for the support width of 65 mm, and the lowest was for the support width of 45 mm, but the Sa was significantly different on the circumference of the holes, depending on the position of the fibers.
- From statistical analysis, the lower mean surface roughness of the drilled hole was obtained for A3 material (wf = 45%), at 45 mm support width. A higher mean surface roughness was achieved for A1 material (wf = 60%) at 65 mm support width.
- The best quality of the holes drilled in twill-based GFRP was achieved by using a lower feed rate of 0.04 mm/tooth, and a support width of 55 mm.

This analysis of hole quality is recommended for GRFP laminates used in aerospace, marine, and automotive industries, especially structural applications. The proposed methodology can be used to evaluate the delamination of the holes drilled in different composite materials manufactured by different processes considering the specific characteristics of each technology and choosing the proper control factors. Thus, the methodology can be used for analyzing other materials such as CFRP or Basalt Fiber Reinforced Polymer (BFRP) which use the same type of material (twill fabric), wf, matrix type, curing, and heat treatment process. Future studies will be focused on the quality analysis of drilling for new composite materials. Generally, when drilling holes in composite materials, especially in the industry where manufacturers want to obtain good surface quality without damage, holes should be drilled at low feed rates and smaller support spacing.

## Figures and Tables

**Table 1:** The properties of the GFRP plates studied.

GFRP	A1	A2	A3	A4
Wf. [%]	60	50	45	64
Thickness [mm]	1.2	1.5	1.7	2.1
Density [kg/m^3^]	1680	1493	1430	1729
Layers used	4	4	4	8

**Table 2:** Control factors and their level for delamination and cutting force analysis of A1–A3 material made by technology 1.

Targets	Support Width, *a*		Weight Fraction, wf		Feed per Tooth, *f_z_*	
	Symbol	Value [mm]	Symbol	Value [%]	Symbol	Value [mm/tooth]
Fd_peel-up	1	45	1	60	1	0.04
Fd_push-out	2	55	2	50	2	0.08
Cutting_force_A	3	65	3	45	3	0.12
	4	75	-	-	4	0.16

**Table 3:** Control factors and their level for roughness analysis of A1–A3 material made by technology 1.

Target	Support Width, *a*		Weight Fraction, wf		Hole_Zone	
	Symbol	Value [mm]	Symbol	Value [%]	Symbol	Value
Sa	1	45	1	60	1	Zone A
	2	55	2	50	2	Zone B
	3	65	3	45		
	4	75	-	-		

**Table 4:** Control factors and their level for delamination and cutting force analysis of A4 material made by technology 2.

Targets	Support Width, *a*		Feed per Tooth, *f_z_*		Fd_Zone	
	Symbol	Value [mm]	Symbol	Value [mm/tooth]	Symbol	Value
Fd Cutting_force *	1	45	1	0.04	1	entry
	2	55	2	0.08	2	exit
	3	65	3	0.12	-	-
	4	75	4	0.16	-	-

* The control factors for cutting force analysis are support width and feed per tooth.

**Table 5:** The percentage contribution ratio for cutting force; The symbol “*” signifies the interaction between factors.

	Cutting_Force_A1–3				Cutting_Force_A4			
Source	Seq SS	F-Value	*p*-Value	PC [%]	Seq SS	F-Value	*p*-Value	PC [%]
Weight_fraction	3805.8	13.59	<0.001	12.52%	-	-	-	-
Support_width	150.6	0.36	0.784	0.5%	707	17.97	<0.001	6.5%
Feed_per_tooth	18,894	44.99	<0.001	62.15%	10,055.7	255.54	<0.001	92.42%
Weight_fraction*Support_width	2103.5	2.5	0.061	6.92%	-	-	-	-
Weight_fraction*Feed _per_tooth	1364.1	1.62	0.198	4.49%	-	-	-	-
Support_width*Feed _per_tooth	1561.5	1.24	0.332	5.14%	-	-	-	-
Error	2519.5	-	-	8.29%	118	-	-	1.08%
Total	30,399	-	-	100%	10,880.7	-	-	100%

**Table 6:** The percentage contribution ratio of the delamination factor for material A1–3 in the case of technology 1.

	Fd_Peel-Up			Fd_Push-Out		
Source	F-Value	*p*-Value	PC [%]	F-Value	*p*-Value	PC [%]
Support_width	10.7	<0.001	4.86	4.74	0.013	5.43%
Weight_fraction	16.64	<0.001	5.03	38.86	<0.001	29.67%
Feed_per_tooth	152.34	<0.001	69.14	40.14	<0.001	45.97%
Support_width*Weight_fraction	4.92	0.004	4.46	1.72	0.173	3.94%
Support_width*Feed _per_tooth	8.84	<0.001	12.03	0.95	0.512	3.25%
Weight_fraction*Feed _per_tooth	1.93	0.131	1.75	2.12	0.101	4.87%
Error			2.72			6.87%
Total			100%			100%

**Table 7:** The percentage contribution ratio of Fd for material A4 in the case of technology 2.

Source	F-Value	*p*-Value	PC [%]
Support_width	2.29	0.147	2.91%
Feed _per_tooth	22.39	<0.001	28.39%
Fd_zone	128.98	<0.001	54.51%
Support_width*Feed _per_tooth	0.75	0.665	2.84%
Support_width*- Fd_zone	4.04	0.045	5.12%
Feed_per_tooth*Fd_zone	1.92	0.197	2.44%
Error			3.8%
Total			100%

**Table 8:** The percentage contribution ratio of Sa for material A1–A3 in the case of technology 1.

Source	F-Value	*p*-Value	PC [%]
Weight_fraction_A	6.39	0.033	8.33%
Support_width	4.98	0.046	9.74%
Hole_area	60.91	<0.001	39.72%
Weight_fraction_A*Support_width	3.76	0.066	14.71%
Weight_fraction_A*Hole_area	2.72	0.144	3.55%
Support_width*Hole_area	10.24	0.009	20.03%
Error			3.91%
Total			100%

## Data Availability

The original contributions presented in the study are included in the article.
